# The cognitive adaptability and resiliency employment screener (CARES): tool development and testing

**DOI:** 10.3389/fpsyt.2023.1254147

**Published:** 2023-09-29

**Authors:** Wilfredo Manuel R. Torralba, Marlyn Thomas Savio, Xieyining Huang, Priyanka Manchanda, Miriah Steiger, Timir Bharucha, María Martín López, Keanan J. Joyner, Rachel Lutz Guevara

**Affiliations:** ^1^TaskUs Inc., New Braunfels, TX, United States; ^2^Department of Psychology, University of California, Berkeley, Berkeley, CA, United States

**Keywords:** content moderator, factor analysis, psychometrics, recruitment, resilience

## Abstract

**Introduction:**

To decrease psychological risk for content moderators, the study initiated the first steps of developing a robust employment screening tool, namely, the Cognitive Adaptability and Resiliency Employment Screener.

**Method:**

The study consisted of three phases with 4,839 total participants.

**Results:**

In Phase 1, a set of 75 items were developed and tested via exploratory factor analysis, yielding three factors (i.e., Psychological Perseverance & Agility, Rumination & Emotional Lingering, and Expressiveness & Sociability) and also reducing the scale to 67 items. In Phase 2 through confirmatory factor analysis, the three-factor structure showed good fit (CFI = .93, RMSEA = .05) and demonstrated sufficient overall reliability. In Phase 3, the convergent validity and divergent validity of the tool were established relative to constructs such as resilience, cognitive control and flexibility, emotion regulation, and optimism.

**Discussion:**

Altogether, the findings revealed that the scale demonstrated good psychometric properties that, pending future studies, may serve as a promising employment screener for content moderators.

## 1. Introduction

When social networking sites made an entry on the internet, the work of reviewing user generated content (UGC) was initially allocated as an additional responsibility to existing staff in support or community teams. As the growth of UGC in quantity and complexity began to resemble a digital “infodemic,” content moderation emerged in parallel as an indispensable and independent professional service. A report by Dixon revealed that in 2022, one million hours’ worth of content was consumed by users in 1 min (1). Since a sizable portion of this content is UGC across platforms, the need to monitor content has become a necessity. Estimates on the exact number of content moderators worldwide may vary or fluctuate. However, as platforms grow from nascent to established, the number of content moderators may grow from 1–5 persons to 1500–10000 persons per platform depending on the volume of UGC (2, 3). As platforms become larger, this number may continue to grow. Content moderators play an important role in the safety of the internet by closely evaluating different types of UGC produced on a given platform, to ensure that they do not violate the decorum and policies of the platform. The types of UGC content moderators review on a daily basis may range from innocuous content to potentially disturbing content such as misinformation, violence, gore, and child sexual abuse material. In order to reduce potential risks of occupational injury (e.g., in this context, secondary traumatic stress or other mental distress) while allowing for efficient recruitment to keep the internet safe, it is important that we develop a thorough yet concise vetting process to recruit best fits, especially those who may exhibit the dispositions and skills to succeed in their jobs without adverse outcomes (4).

The primary guiding principle in recruiting and training for content moderation needs to be the maximum alignment between the candidate and the job. The ability to learn and execute nuanced rules enables the content moderator to swiftly determine the course of action for each post being reviewed. Secondly, the capacity to self-regulate emotions and maintain composure across situations helps moderators function optimally. Additionally, it is important to provide adequate support to protect the psychological health of content moderators who are exposed to potentially disturbing content. A recommended practice is to hire applicants who either at the outset show evidence of preexisting likelihood of resiliency toward content exposure or exhibit cognitive and emotional traits that may increase resiliency with appropriate training and intervention. Company-led psychological safety programs may then be able to best serve and protect persons who exhibit such potential from the point of hiring. Although a few Western tertiary institutions (e.g., Stanford University, Griffith College) recently started offering courses on content moderation, these tend to be designed for managers, executives, or engineers rather than frontline content moderators. Since no formal qualification or training is provided for this frontline role outside the industry, it becomes vital to pursue a balanced assessment measuring the aspirant’s existing skill set and readiness for content moderation (4). Ensuring person-job fit is a highly practical approach to set candidates up for success in content moderation as well as protect them from the potential fallouts of UGC.

Two common methods that recruiters depend on to assess these qualities include educational review and interviews. The subjectivity and non-comprehensiveness inherent in such hiring modalities may arguably be a factor for the oft-cited role conflict, dissatisfaction and attrition seen among content moderators (5). Early in the selection process, time-saving and low-cost methods are needed to help differentiate those whose dispositions may put them at particular risk of negative impacts related to the standard job duties of content moderation. This way the pool of applicants who are a good match for this kind of work is condensed for a more thorough and specialized review.

A promising solution in large-scale talent acquisition for content moderation may be the use of psychometric tests. Historically, psychometrics allowed for cost-effectively shortlisting a strong cohort of candidates (6). In fact, in similar professions that involve the risk of trauma on the job (e.g., military), the implementation of screening protocols before deployment was routinely adopted and observed to reduce instances of physical and behavioral health problems on the job (7). Although not imminently life-threatening, content moderators’ exposure to unfiltered content may result in changes to personal beliefs or worldview and in extreme instances potentiate negative emotional states akin to trauma (2). Therefore, it is important to adopt similar practices of incorporating psychometrics in the screening process.

There is, however, a dearth of contemporary psychometric measures that gauge the specific qualities necessary to sustain and succeed in content moderation. Although broad employment readiness assessments do exist outside of content moderation, they are largely generic in nature, and applicable to most business process outsourcing jobs (examining language comprehension, computer skills, etc.). Generalized assessments are inadequate to assess the cognitive and emotional attributes needed for the content moderation work. Likewise, personality tests–although highly favored by multinational companies during hiring–have been shown to poorly reflect actual job performance when used alone (8). Adding more assessments to support personality measures, however, only increases mental fatigue for the respondent.

Given the absence of assessments in the public domain to succinctly assess the cognitive and emotional qualities that protect against the potential dangers or challenges inherent in content moderation, this study was undertaken to create such a tool for screening future content moderators. Although it is important to screen for candidates who may likely be resilient at the outset, it is equally important to assess cognitive and emotional qualities that may help maintain and boost resiliency as employees start and continue in their moderation job. Since measures on resiliency already exist (9, 10), we aimed to develop a scale that focuses on cognitive and emotional qualities that are essential for developing and maintaining resilience. Of note, while it is imperative that the screening tool methodically evaluate psychological domains, the study made conscious efforts to ensure that the use of the tool does not culminate in labeling or diagnosing candidates.

As part of the first few steps of constructing a comprehensive multidimensional scale, three study phases were carried out in this present investigation. The first phase involved the construction and initial testing of the potential item set, including exploratory factor modeling among other techniques. In the second phase, the item set was further refined to present a more robust factor model relevant to content moderation using confirmatory methodologies. The third phase involved establishing the convergent and divergent validity of the finalized item set. These three phases were critical for future studies to build on the current findings and to examine its predictive utility in risk for adverse outcomes and success on the job.

## 2. Phase 1

To understand what cognitive or emotional constructs the employment screener should assess, a literature review was conducted to examine what qualities may help an individual succeed in content moderation and reduce the likelihood of workplace psychological injury. Overall, the review found that the literature on content moderation specifically was scarce. From published literature, one study suggested that resiliency, the ability to bounce back from stressful situations, may be a protective factor against negative psychological outcomes for content moderators (11). This finding is supported by the broader literature on how highly resilient individuals may be able to better manage their stress than those with low resiliency, even during high-stress situations (12, 13). Highly resilient individuals have also been found to exhibit lower rates of mental illnesses (14). Content moderators may often be exposed to stress-inducing content, therefore emotional and cognitive processes associated with resiliency are important constructs to consider in the employment screener.

Literature suggests that cognitive and psychological constructs such as emotion regulation, optimism, and grit may play a protective role for the psychological health of content moderators. Emotion regulation, the processes of influencing how one’s emotions are experienced and expressed (15), has been found to be an important mediator for psychological and physical health (16, 17). For example, evidence suggests that emotion regulation can mediate burnout levels (18, 19), particularly relevant for content moderation. Furthermore, emotion regulation can strengthen or weaken the fear response and negative emotion; deficits in emotion regulation were prospectively associated with the development of anxiety symptoms longitudinally (20, 21).

Cognitive factors may also be critical qualities to consider for content moderation. Cognitive control, the ability to regulate, coordinate, and manage thoughts to be aligned with goals (22), plays an important role in creative problem solving. Content moderators must apply intricate and quickly evolving policies to unique and nuanced situations, requiring constant problem solving. Meanwhile, cognitive flexibility, the ability to adjust one’s behavior depending on the need of the environment (23), is crucial for modulating negative psychological and physical outcomes, which in turn would improve performance (24, 25). Despite appearing seemingly different, the two constructs were theorized to complement each other (26) and may together lead to a successful performance in content moderation.

Two additional qualities, grit and optimism, may further amplify a content moderator’s performance (27, 28). Grit, defined as perseverance and passion for long-term goals, has been found to produce long-term effects in maintaining engagement in projects or the job (29), and thus may aid in employee retention in content moderation lines. Similarly, optimism has been shown to act as a buffer against the possibility of diminishing self-regulation as stress builds up (30). Additionally, optimism can predict the overall cognitive, emotional, and physical engagement, which in turn predicts performance (31). In content moderation, it is important to remain engaged while performing consistently, and so assessing job candidates’ optimism may help predict their work performance.

In addition to protective factors, literature review also revealed several important risk factors to consider for the employment screener. Specifically, three cognitive and emotional constructs may be especially relevant to content moderation: impulsivity, neuroticism, and the fear and worry response. Impulsivity is often defined as performing an action without a certain form of control or perseverance (32). Research has shown that impulsivity is consistently associated with negative psychological and professional outcomes, such as addiction (33–36) and suicidal behavior (37). Furthermore, another feature of individuals with high impulsivity is the tendency to choose immediate rewards over delayed rewards. This may further lead to risky choices due to the immediacy of reinforcement (38, 39). Additionally, individuals with high impulsivity may exhibit poorer planning capabilities, and manifest frustration and stress as responses to unusual situations (39). Studies have also shown that high impulsivity may interfere with the protective effects of resiliency (40) as well as decrease the effects of wellness intervention (41).

Relatedly, neuroticism, a personality factor that involves a pattern of negative emotions and worry (42, 43) may be a risk factor for negative outcomes among content moderators. Research has shown that patterns of negative emotions and worry could jeopardize overall health (35, 43, 44). Individuals with elevated levels of neuroticism may be prone to environmental stress as they tend to view stressful situations as threatening (44). Given the potential stressful nature of content moderation (e.g., exposure to graphic content, changes in platform policies), it is critical to select candidates with low neuroticism to ensure content moderator health.

Lastly, fear and worry responses may also predict health risks for those engaging in content moderation work. Fear is an emotional response to perceived danger that is usually accompanied by distress (45), while worry is defined as a form of negative expectation toward usual concerns (46). Although certain levels of fear and worry response may be normal and adaptive (47), experiencing fear and worry at an elevated level may pose health risks as well as predict underperformance in content moderation. Research has shown that individuals manifesting greater fear after an injury were less likely to return to physical activity (48). Similarly, fear of reinjury and perceived uncertainty have been shown to prevent construction workers from returning to work (49). It is possible that fear of psychological injury may reduce a content moderator’s ability to succeed. Relatedly, individuals with high worry may exhibit higher intolerance of uncertainty (50). In content moderation, there can be a level of uncertainty about the nature of the content one may be exposed to on any given day. Therefore, it is important to identify and hire moderators who are most likely able to cope with uncertainty, with appropriate support.

Based on the above literature review of protective and risk factors in cognitive and emotional domains, as well as researcher clinical expertise, an initial set of 187 questions was created for the new tool. The items focused on resiliency, emotion regulation, cognitive factors, optimism, grit, impulsivity, neuroticism, and the fear and worry response. To finalize the question items to be tested, the researchers evaluated each of the 187 items independently based on face validity (i.e., whether each item is relevant to potential protective and risk factors for content moderation as discussed above), cultural relevance, and ease of comprehension (i.e., no more than 8^th^ grade reading level). Next, by means of a live voting session, only items that reached consensus among the researchers were retained, resulting in a total of 75 items (Appendix 1). The goal of the first phase was to explore the potential factor structure of the employment screener and to examine its internal validity by conducting a series of exploratory factor analyses (EFAs).

### 2.1. Method

#### 2.1.1. Study sample and procedures

The study procedures were approved by the ethics review board at TaskUs Inc., which was chaired by a mental health researcher from an external research and academic institution. Additionally, procedures complied with the code of conduct, legal regulations, and ethical guidelines set out by TaskUs Inc. The research team is an independent unit within the organization that shares no direct relationship with or oversight of content moderation teams or projects at the company. These efforts helped to minimize potential conflicts of interest. There was no report of adverse outcomes during the course of the study.

As a part of the application process for a content moderator position at TaskUs, all candidates were invited to complete the employment screener for research purposes between December 2021 and January 2022. Participants were assured that their decision on whether or not to complete the research section questions as well as their answers in the research section would not be a factor in their selection process. Consistent with the geographic distributions of content moderators for the industry as well as TaskUs operations at the time of data collection, almost all candidates resided in the Philippines. Therefore, this study focused on identifying the factor structure and examining the validity of the employment screener among Filipino candidates. A total of 3,356 respondents completed the employment screener electronically. The survey took approximately 20 min. Of note, we were unable to collect information on age and gender due to legal regulations and company non-discriminatory policies governing recruitment.

#### 2.1.2. Measures

The Cognitive Adaptability and Resiliency Employment Screener (CARES) was created from a pool of 75 items in an employment screener developed by the authors at the company to gauge the cognitive and psychological qualities essential to content moderation (Appendix 1). A total of 24 items focused on emotion regulation (e.g., “I prefer not to tell others what I am feeling,”), 10 items on cognitive factor (e.g., “I have a difficult time adjusting to last minute changes”), 10 items on neuroticism/impulsiveness (e.g., “I do not let myself become ‘stuck’ on past events”), 10 items on optimism (e.g., “I maintain positivity even when others around me are not”), 7 items on grit (e.g., “Difficulties do not discourage me”), and 14 items on fear and worry response (e.g., “It is easy for me to let go of worrisome thoughts”). Each item is rated on a 7-point Likert scale from 0 (“Strongly Disagree”) to 6 (“Strongly Agree”). For descriptive statistics of each item, please see Appendix 2. Of note, the CARES tool did not contain any graphic images or text that could potentiate psychological concerns (see Appendix 1). Akin to many other survey studies involving psychological measures, participation in this study had minimal risk.

#### 2.1.3. Analysis plan: EFA model specification

In the Phase 1 data (n = 3,356), an EFA with the 75 potential CARES items was conducted using the ‘psych’ package in R (v2.3.3) (51), using maximum likelihood estimation with promax rotation. To determine the number of factors to extract, a combination of examination of the scree plot (52) and parallel analysis (53) was used. Furthermore, the results of this initial EFA were considered in light of the practical use of the intended final measure – that is, there was consideration of the balance of model complexity/parsimony for purpose of minimizing the number of items that adequately reflected the intended target constructs, as the measure will need to be fairly brief to be widely adopted. After the number of total factors were selected, item-level characteristics were examined to determine which items to retain in a final measure. Following recent recommendations for refining “clean” factors (54), items that did not load on any factor ≥ |0.4| or loaded on more than one factor ≥ |0.4| were excluded from further consideration. With these modifications, the items loading onto each factor were initially tested for internal consistency for use as a sum score (i.e., as would be used in practice), using Cronbach’s alpha (α) and MacDonald’s omega (ω).

### 2.2. Results and discussion

**Figure 1:**
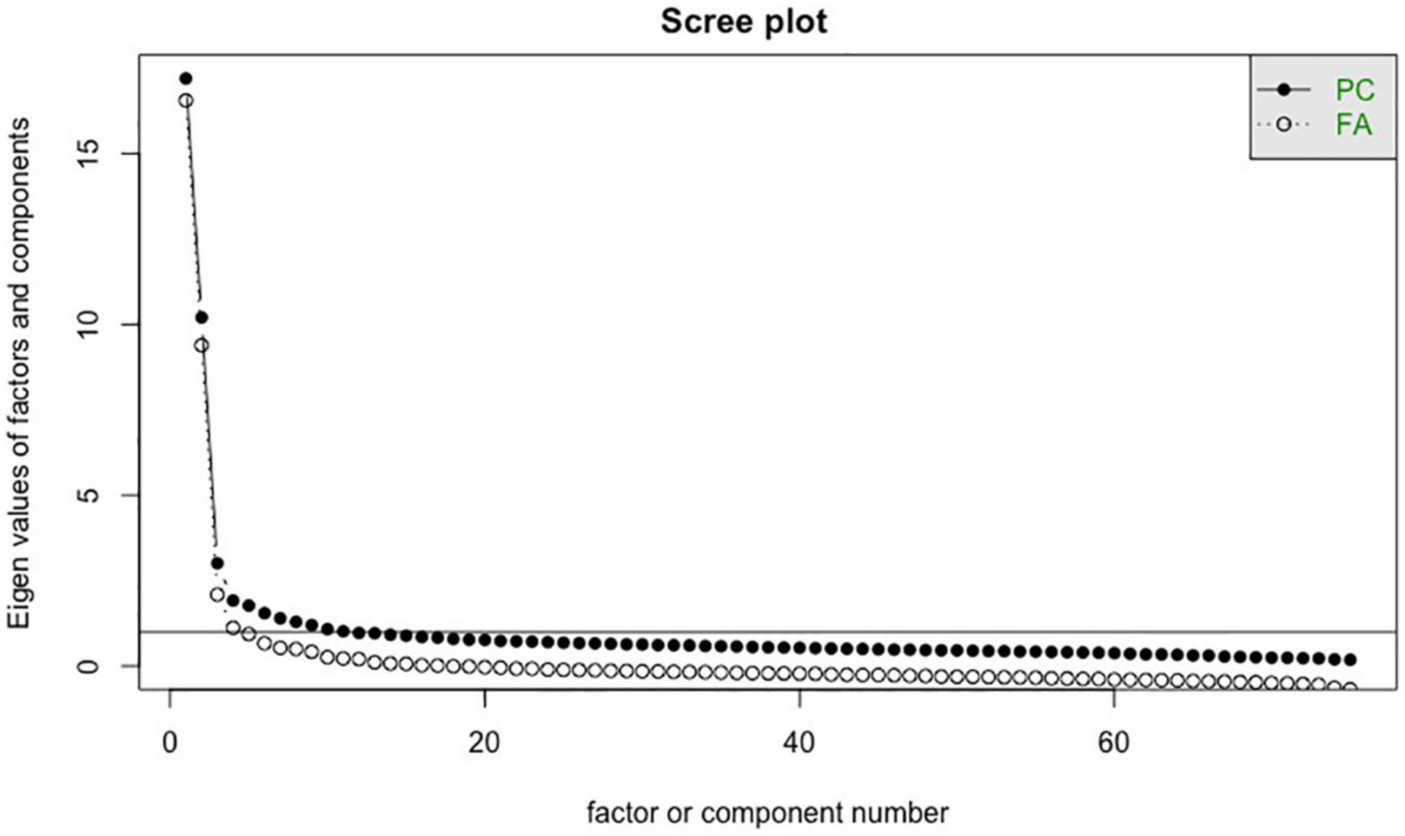
Scree plot for retaining factors (exploratory factor analysis). PC, principal component; FA, factor analysis.

Based on Kaiser’s criterion (i.e., eigenvalues larger than 1) (55), the latent structure of the CARES was best explained by 12 factors (Figure 1) and a parallel analysis indicated eight factors. However, inspection of the scree plot suggested the retention of only three factors (Figure 1). As such, we compared the three-factor model with these other models. Unlike the 12-factor model, the three-factor model produced a cleaner version of the test with no items with cross loadings. Additionally, two of those factors in the 12-factor structure did not evidence any indicators without cross-loadings, leaving no unique items for those factors. Given the desire for simple structure and an abbreviated inventory for practical use, the three-factor model was selected. In the three-factor model, items that still showed cross loadings of |0.40| on multiple factors were removed (54). The final three-factor model retained 67 items, explaining approximately 38% of the total variance of all items. The three factors also functioned well as sum scores as indicated by high internal consistency reliability estimates (first factor: *α* = 0.96, *ω* = 0.97; second factor: *α* = .94, *ω* = .94; third factor: *α* = .77, *ω* = .87). For descriptive statistics of each CARES item, please see Appendix 3.

After evaluating the content of items on each of the factors, we named Factor 1 as Psychological Perseverance and Agility (PPA), Factor 2 as Rumination and Emotion Lingering (REL), and Factor 3 as Expressiveness and Sociability (ESc). PPA includes 30 questions, with REL including 30 questions and ESc including seven questions (see Appendix 4 for details). This led to the next phase wherein we tested the three-factor model using CFA to *a priori* test the fit of the hypothesized factor structure.

## 3. Phase 2

The major aim of Phase 2 was to evaluate the confirmatory fit of the proposed structure of the CARES by conducting a Confirmatory Factor Analysis (CFA). We utilized CFA to test the latent model in a confirmatory framework. In contrast to the EFA in Phase 1 in which all items were permitted to freely load onto any factors, the model parameterization was specified *a priori* for the CFA in Phase 2 based on the results of Phase 1. That is, items determined to be indicators of PPA were only permitted to load onto PPA in the CFA, likewise for REL and ESc. This approach allowed us to empirically test whether PPA, REL, and ESc are distinct yet related constructs potentially associated with content moderator health and success.

### 3.1. Method

#### 3.1.1. Study sample and procedures

Akin to Phase 1, Phase 2 procedures were approved by the ethics review board at TaskUs Inc. Identical participant recruitment procedures were followed in Phase 2 as Phase 1, where candidates for content moderator positions provided informed consent prior to complete a survey including question items from the CARES. Responses were collected in February 2022. Similar to Phase 1, all responses came from the Philippines (*N* = 956). Again, we were unable to collect information on age and gender due to laws and regulations as well as the non-discriminatory practices of the company.

#### 3.1.2. Analysis plan: CFA model specification

A CFA with the 67 retained CARES items was conducted using the ‘*psych*’ package in R (v2.3.3) (51). Considering that each model fit index is associated with unique strengths and weaknesses, multiple fit indices were used to evaluate the models: (1) chi-square (χ^2^); (2) the standardized root-mean-square residual (SRMR); (3) the comparative fit index (CFI); (4) the Tucker–Lewis index (TLI); and (5) the root-mean-square error of approximation (RMSEA). Both χ^2^ and the SRMR are indices of absolute model fit, whereas the CFI and the TLI are indices of comparative model fit, and RMSEA also accounts for model parsimony. Prior research indicated the following cutoff criteria for the present study: CFI and TLI values around 0.90, and SRMR and RMSEA values ≤0.08 represent the lower bound of potentially acceptable fit (56, 57).

### 3.2. Results and discussion

**Figure 2:**
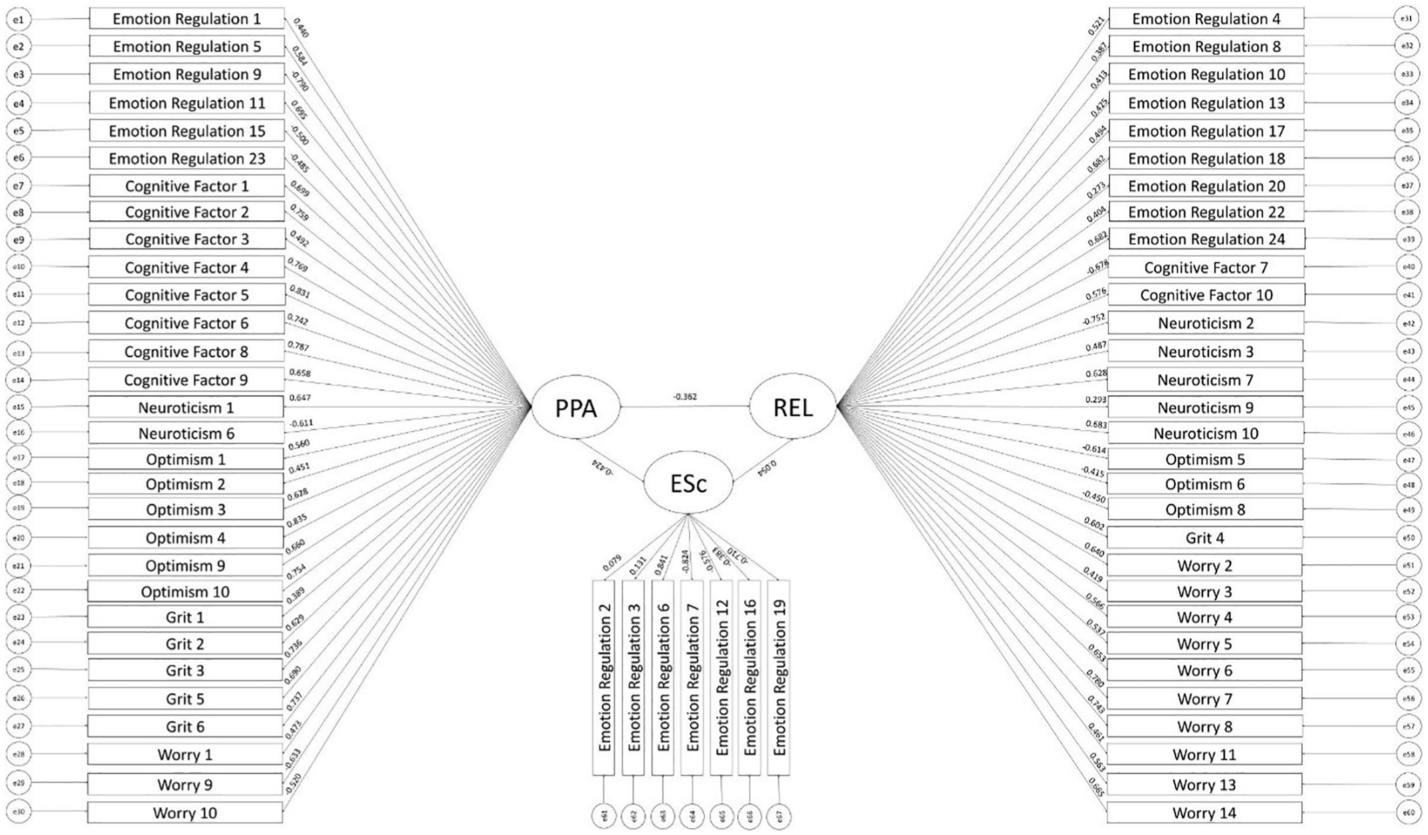
Three-factormodel (confirmatory factor analysis). PPA, Psychological Perseverance & Agility; REL, Rumination and Emotional Lingering; ESc, Expressiveness and Sociability.

Using diagonally weighted least squares (DWLS) estimation due to the ordinal nature of the items, the hypothesized three-factor model fit the data well, c2(2141) = 7360.53, *p* < 0.001, CFI = .928, TLI = .926, RMSEA = .05, SRMR = .07 (Figure 2). Factor loadings were generally moderate-to-high and even across factors (PPA: mean *λ* = 0.64; REL: mean *λ* = .55; ESc: mean *λ* = .51). Replicating Study 1, the three factors also functioned well as sum scores as indicated by high internal consistency reliability estimates in Study 2 as well (PPA: *α* = .96, *ω* = .96; REL: *α* = .93, *ω* = .94; ESc: *α* = .76, *ω* = .87). Additionally, the average variance extracted (AVE) for the factors reflect varying levels of variance the latent construct accounts for in the manifest indicators. Specifically, PPA exhibited the highest AVE of. 39, while REL and ESc demonstrated more moderate AVE of .30 and .33 respectively. In the context of the hierarchical testing of multitrait-multimethod (HTMT) analysis, the correlations between the factors all support good discriminant validity (58) (Appendix 5).

## 4. Phase 3

The goal of Phase 3 was to investigate the convergent and divergent validity of the CARES in relation to conceptually related and distinct constructs in the literature. Establishing convergent and divergent validity is a crucial step in the test development process to help demonstrate that the newly designed test is unique when compared with existing tests. Existing tests that seemingly would correlate (either positively or negatively) with the current factors derived from the new inventory. The *a priori* criteria used in the current work for correlation coefficients that would be considered in establishing convergent and divergent validity are 0.00–0.19 (negligible association), 0.20–0.49 (low association), 0.50–0.69 (moderate association), 0.70–0.85 (high association) and 0.86–1.00 (very high association) (59), as recommended in psychometrics literature [e.g., (60, 61)].

### 4.1. Method

#### 4.1.1. Study sample and procedures

Phase 3 procedures were identical to Phases 1 and 2 in that data were gathered from job candidates who had applied for content moderator positions. The responses were collected from April to May 2023. Similar to Phases 1 and 2, all responses came from the Philippines (*N* = 527).

#### 4.1.2. Measures

Similar to CARES, none of the additional tools administered in this phase contained graphic content that could potentiate psychological concerns. Participation involved very minimal risk, akin to many other surveys involving psychological measures. The current study took approximately 30 min for participants to complete.

Because of legal regulations in the recruitment process, the current study was unable to include clinically diagnostic measures that assess prior psychological injury/trauma among respondents. It is also important to note that as the survey was being administered at the point of job recruitment, we did not include any measures that assess for potential psychological injury/trauma as a result of reviewing graphic content as candidates had not yet been exposed to job-related content.

##### 4.1.2.1. Connor Davidson Resilience Scale (CD-RISC 10)

The CD-RISC 10 (62) is a scale that measures resiliency or how well equipped a person is to “bounce back” after stressful events, tragedy, or trauma. Resiliency gives us the ability to thrive in the face of adversity (62). It contains items such as “I am able to adapt when changes occur” and shows excellent internal consistency in the current sample (Cronbach’s *α* = 0.91).

##### 4.1.2.2. Cognitive control and flexibility questionnaire (CCFQ)

The CCFQ (26) measures the ability to adapt to changes and has been associated with goal-oriented behaviors including creativity, problem solving, multi-tasking, and decision making. It contains two subscales, namely: Cognitive Control Over Emotions, and Appraisal and Coping Flexibility. Cognitive Control over Emotions focuses more on the executive control of emotions, stress, and challenges. It contains items such as “It is easy for me to ignore distracting thoughts.” Appraisal and Coping Flexibility on the other hand focuses more on the regulation and selection of coping that alleviate the stress of control. It includes items like “I manage my thoughts or feelings by reframing the situation.” The scale showed excellent internal consistency in the current sample (*α* = 0.92).

##### 4.1.2.3. Emotion Regulation Questionnaire (ERQ)

ERQ (63) is a 10-item scale designed to measure respondents’ tendency to regulate their emotions. The questionnaire categorizes two types of emotional regulation: Cognitive Reappraisal and Expressive Suppression. Cognitive Reappraisal is a type of coping where reframing of situations is quite common while Expressive Suppression is usually withholding any type of emotion. It contains questions like “I control my emotions by changing the way I think about the situation I’m in.” for Cognitive Reappraisal, and “When I am feeling negative emotions, I make sure not to express them.” for Expressive Suppression. The scale was found to have good internal consistency in the current study (*α* = 0.74).

##### 4.1.2.4. Revised Life Orientation Test (LOT-R)

LOT-R (64) is a 10-item scale that measures optimism. It is defined as the perception about the positive and negative expectation about the future. Showing that pessimism, the opposite of optimism is in a continuum. The scale consists of true items and filler items. For example, “In uncertain times, I usually expect the best.” as a true item and some filler items like “It’s easy for me to relax.” In scoring, the filler items will not be considered to be part of the total score. Higher scores indicate optimism and lower scores indicate pessimism. The test somehow showed poor internal consistency (*α* = 0.57).

##### 4.1.2.5. Dunn Worry Questionnaire (DWQ)

DWQ (65) is a 10-item scale that assesses general worry. The measurement was good in informing wide ranges of worry and is sensitive to change. During the development of the scale, another scale emerged but for the purposes of this study the 10-item scale was the only one considered to be part of the research. The scale consists of questions pertaining to worry like “Worry has caused me to feel upset” and “I have been worrying even though I did not want to.” The scale demonstrated excellent internal consistency (*α* = 0.90).

##### 4.1.2.6. Berkeley Expressivity Questionnaire (BEQ)

BEQ (66) is a 16-item questionnaire that measures emotional expression. As theorized by Gross and John emotions only suggest how we act therefore expressing emotions may be different for individuals. The scale has 3 subscales namely: Negative Expressivity, Positive Expressivity, and Impulse Strength. Negative expressivity pertains to the expression of negative emotions while positive expressivity is for expression of positive emotions. Impulse strength pertains to the ability to control impulses once the emotions are present. Items about negative expressivity include “It is difficult for me to hide my fear,” positive expressivity “Whenever I feel positive emotions, people can easily see exactly what I am feeling.,” and impulse strength “My body reacts very strongly to emotional situations.” The questionnaire showed good internal consistency (*α* = 0.80) within the current sample.

### 4.2. Results and discussion

As seen in the intercorrelations among variables in Phase 3 (Table 1), the three CARES factors showed patterns of convergent and divergent validity with respect to the variety of external criteria. For the purposes of this analysis, we considered moderate to high association to establish good convergent and small to zero association for divergent validity (60, 61). As seen in Table 1, PPA showed good convergence with resilience (*r*(524) = .62) and cognitive control and flexibility (*r*(524) = .75). With respect to REL, it showed negative correlations with resilience (*r*(524) = −.52), cognitive control and flexibility (*r*(524) = −.71), and a positive correlation with worry (*r*(524) = .66) and impulse strength (*r*(524) = .48), a subscale of the BEQ. In terms of ESc, the associations were overall weaker compared with PPA and REL. The strongest observed correlation was between ESc and cognitive control and flexibility (*r*(524) = −0.37). Nonetheless, ESc demonstrated consistent (albeit smaller) correlations across different measures.

**Table 1:** Convergent and divergent validity.

Variables	PPA	REL	ESc
CD-RISC 10	0.62	−0.52	−0.26
CCFQ	0.75	−0.71	−0.36
Cognitive control over emotions	0.62	−0.75	−0.37
Appraisal coping flexibility	0.73	−0.51	−0.27
LOT-R	0.37	−0.41	−0.29
DWQ	−0.44	0.66	0.30
BEQ	−0.10	0.40	−0.08
Negative expressivity	−0.34	0.44	0.07
Positive expressivity	0.25	−0.01	−0.31
Impulse strength	−0.16	0.48	−0.06
Cognitive reappraisal	0.51	−0.35	−0.18
Expressive suppression	0.08	0.06	0.28

PPA, Psychological Perseverance & Agility; REL, Rumination and Emotional Lingering; ESc, Expressiveness and Sociability; CD-RISC 10, Connor Davidson Resilience Scale 10; CCFQ, Cognitive Control and Flexibility Questionnaire; LOT-R, Life Orientation Test Revised; DWQ, Dunn Worry Questionnaire; BEQ, Berkeley Expressivity Questionnaire; ERQ, Emotion Regulation Questionnaire.

In terms of divergent validity, PPA showed divergent validity with BEQ overall as well as its subscale Expressive Suppression (*r*(524) = −.10; Table 1). Similarly, REL was uncorrelated with two BEQ subscales, namely, Positive Expressivity (*r*(524) = −.01) and Expressive Suppression (*r*(524) = .06). Lastly, ESc was uncorrelated with two other BEQ subscales: Negative Expressivity (*r*(524) = .07) and Impulse Strength (*r*(524) = .06).

## 5. General discussion

The purpose of the study was to complete the first few foundational steps to develop a screening tool for potential content moderators. Given the dearth of literature on the content moderation profession, this preliminary study set out to identify factors that are relevant to the job and help set candidates up for success in this line of work. The initial literature review revealed themes related to resilience, productivity, cognitive capacities and emotional strength as critical for content moderation work. Items generated on these themes were tested using exploratory factor analysis (EFA) and confirmatory factor analysis (CFA) for a robust screener. The resulting 3-factor scale with 67 items demonstrated adequate reliability and validity when tested on large samples from the Philippines.

The three factors of CARES represent promising protective and risk factors that may be uniquely impactful for content moderation success. Furthermore, PPA, REL and ESc reiterate the significance of cognitive and emotional traits that contribute to making an individual resilient. The first factor PPA involves items regarding the healthy management of stress, self-regulation, and perseverance in tasks which collectively represent the capacity for adaptation. These cognitive items are necessary for productivity in content moderation such as attention, multitasking and startle recovery. Research by Ikebuchi et al. (67) revealed that improvement in cognitive functioning resulted in longer tenure on the job even among individuals with severe mental illness. For content moderators, better cognitive functioning would likely predict better work output in a fast-paced and potentially disturbing workflow. The second factor REL includes items such as uncontrolled worry and challenges with regulating one’s emotions that have costs at the individual level (e.g., emotional exhaustion, job satisfaction) (68). Additionally, the lingering of emotions or the inability to process them beyond a reasonable time frame may be debilitating. As exposure to emotionally stirring, graphic material is a routine part of content moderation, the inability to process lingering emotions could potentially jeopardize adaptive functions such as adjustment in behavior and motivation (69). The last factor ESc includes items assessing how one expresses and shares their emotions. As a content moderator, it is important to appropriately share the potential impact of content on their emotional health so that they can receive prompt intervention. Together, the three factors offer a holistic measurement of resilience as relevant for content moderators’ well-being.

The present investigation represents one of the first few research efforts to create a scale that can be used as a content moderation employment screener. Future studies in other samples need to establish the replication of factor structure as well as the predictive utility of the CARES on reduced psychological risk and increased job success; one or multiple factors of the CARES has the potential to be used to improve the content moderation recruitment process as well as the moderator experience. CARES may serve as a time- and cost-saving self-report scale that enables filtering of the talent pool to process strong-fit candidates further along the recruitment process. In comparison with more general psychometric tests, CARES may function as a specialized instrument for use with content moderators, who represent a growing population in the technology workforce. Nevertheless, the dimensions of CARES may also be relevant in recruitment for various other frontline work besides content moderation. In fact, any profession that requires cognitive and emotional agility (e.g., first responders, social workers, customer-facing personnel) may benefit from prior screening. Tools like CARES not only provide evidence on candidate fitness but also offer potential recruits an invaluable opportunity for self-evaluation with respect to the job demands. Prospective studies are encouraged to examine the usefulness of CARES in other professional lines, though it is important to consider whether such screening can be included per local employment laws.

There are global concerns about occupational trauma risk in this type of work (2, 70), accompanied with calls for better protection of moderators’ well-being (71, 72). Arguably, CARES may address concerns about trauma on the work by prioritizing a preventative approach that screens individuals who may be at increased risk or whose preferences may not align with the job functions even before they are hired for a content moderation role. Pertinently, the addition of CARES in hiring may instill the perception of psychological safety from the very outset in the candidates’ experience with the organization. Data gathered by gauging cognitive and emotional qualities can also help inform and customize psychological safety programs for meaningful adjustment and growth in content moderation among selected recruits.

Despite the promising nature of the CARES and the contribution of the present investigation to the literature, it is important to consider the current findings in the context of the study limitations. First, while the CARES has demonstrated adequate reliability and validity, establishment of its prospective predictive validity is needed prior to using it as an employment screening tool. A follow-up study in new samples is also critical to replicate the factor structure and establish tool standardization (including the development of norms and cut-off thresholds). There is precedence in the field of personality and psychological research to include factors with AVEs lower than 0.50 (73–75). In addition, while factor loadings are generally ideally higher, it is usually noted that the acceptability of factor loadings should not be solely determined by a specific threshold value. Instead, it is good practice for researchers to consider multiple factors, like the theoretical significance of the factor loading, the sample size, the reliability of the measurement instrument, and the overall model fit indices (76, 77). The present investigation, therefore, provides the steps necessary for a follow-up study to confirm or revise the factor structure. Thirdly, as the current sample was exclusively based in the Philippines, future phases must consider including other nationalities to increase the relevance of the tool for regions where content moderation is a growing profession such as South Asia, Europe, Middle East, and Africa. These efforts could aid the development of credible translations and adaptations of the tool in addition to the existing English form.

Thirdly, due to local employment laws, we were unable to include demographics questions such as age and gender in the survey. Therefore, we were unable to examine how the psychometric properties may vary depending on demographic characteristics. Future studies may aid in this endeavor when possible such as through demonstrating measurement invariance with respect to demographic variables like age, gender and culture to avert any biases in hiring. Lastly, the present sample, although involving potential recruits who were not yet a part of the company, was sourced only at TaskUs. Given that companies have strict data sharing regulations, it becomes challenging to recruit participants from different companies for a shared study. Partnering with academic universities for multi-company studies may serve as a possible workaround. Despite the aforementioned shortcomings, the current study adopted a rigorous psychometric tool construction approach. It serves as a necessary foundation for the development and standardization of a screening instrument for content moderation jobs.

To summarize, the present investigation found that the CARES showed good psychometric properties through exploratory factor analysis and confirmatory factor analysis. Additionally, it demonstrated good convergent and divergent validity when correlated with relevant measures. Together the findings suggest that the CARES, pending longitudinal follow-up studies, may serve as a viable employment screening tool for content moderation. If future studies support its predictive validity, the CARES has the potential to help identify individuals who may be less likely to develop adverse outcomes and more likely to succeed at the job. When content moderator health and success is prioritized, online platforms and communities may be better protected, leading to a safer experience for online users.

## Data availability statement

The raw data supporting the conclusions of this article will be made available by the authors, without undue reservation.

## Ethics statement

The studies involving humans were approved by Ethics Review Board at TaskUs Inc. The studies were conducted in accordance with the local legislation and institutional requirements. The participants provided their written informed consent to participate in this study.

## Author contributions

WT: Methodology, Writing – original draft, Writing – review & editing, Formal analysis, Investigation, Project administration. MaS: Methodology, Writing – original draft, Writing – review & editing. XH: Methodology, Resources, Supervision, Writing – original draft, Writing – review & editing. PM: Data curation, Formal analysis, Resources, Software, Validation, Writing – review & editing. MiS: Conceptualization, Methodology, Project administration, Resources, Supervision, Writing – review & editing. TB: Conceptualization, Data curation, Methodology, Writing – review & editing. ML: Formal analysis, Writing – review & editing, Validation. KJ: Methodology, Writing – review & editing, Formal analysis. RG: Conceptualization, Methodology, Project administration, Resources, Supervision, Writing – review & editing.
