# Supplementary material for: The cognitive adaptability and resiliency employment screener (CARES): tool development and testing
Source: Front Psychiatry. 2023 Sep 29;14:1254147. doi: 10.3389/fpsyt.2023.1254147 (PMC10570752; doi:10.3389/fpsyt.2023.1254147)
Supplement: Supplementary file 1 [file Data_Sheet_1.zip › Appendix 1_CARES_Original 75 items.docx]

**CARES for EFA (75 items)**

The items analyzed with EFA included 24 items on emotional regulation (e.g., “I prefer not to tell others what I am feeling,” “It is difficult for me to not get overwhelmed”), with 10 items centering on cognitive control and flexibility (e.g., “I have a difficult time adjusting to last minute changes,” reverse; “I am able to set aside unwanted thoughts”), 7 on grit (e.g., “Difficulties do not discourage me”), 10 items focus on optimism (e.g., “I maintain positivity even when others around me are not.”), 10 items focused on impulsiveness and neuroticism (e.g., “I do not like working on tasks that I do not find rewarding.”), and 10 items focused on fear and worry response (e.g., “It is easy for me to let go of worrisome thoughts,” reverse). Each item is rated on a 7-point Likert scale from 0 (“Strongly Disagree”) to 6 (“Strongly Agree”).

Emotion Regulation

1. It only takes a few moments to calm myself following an argument.
2. I prefer not to tell others what I am feeling.
3. I prefer not to tell others what I am thinking.
4. I have a tough time speaking up for myself.
5. I tend to think more logically instead of emotionally.
6. I would consider myself as someone who shares feelings openly.*
7. I am very open and inform others of my current feelings.
8. When an opportunity is given to me to share how I feel, I prefer not to.
9. I am able to remain calm following any stressful situation.
10. I become easily overwhelmed when provided too many tasks.
11. I quickly recover from being startled/ alarmed.
12. I am a person who is highly expressive about my emotions.
13. Work interruptions tend to upset me.
14. I worry more about others' needs to the point that I disregard my own.
15. I care about how my decisions will impact others.*
16. I have a tendency to express my emotions immediately.
17. I find it difficult to relax after viewing disturbing content on my television or computer.
18. Whenever I feel excited, it is difficult for me to focus.
19. It is easy for me to express my emotions.
20. It is difficult for me to not get overwhelmed.
21. I am highly sensitive to others emotions or current mood.
22. I consider myself as an emotional person.
23. I rely on facts over intuition.*
24. I tend to remain upset for long periods of time following an argument.

Cognitive Factor

1. I am able to prioritize and focus on important tasks.
2. When I feel sad, I can still do the tasks that need to be done.
3. After I resolve a problem, I do not obsess about it afterwards.
4. I am comfortable multitasking.
5. I am able to set aside unrelated thoughts while focusing on a current task/conversation.
6. I am able to apply past successful strategies to current difficulties.
7. I have a difficult time adjusting to last minute changes.*
8. I am flexible and comfortable with rapid changes.
9. If I do not succeed on my first attempt, I find other solutions.
10. I cannot switch tasks when I am in deep concentration.

Neuroticism/Impulsiveness

1. I do not let myself become “stuck” on past events.
2. I get distracted by my thoughts.*
3. I only work on tasks that I find rewarding.
4. I avoid all situations that make me feel anxious.
5. I know when to leave a situation when it becomes overwhelming. *
6. I can focus on my work even when I am in a noisy environment.*
7. I get bored doing repetitive tasks.
8. If I see a notification on my phone, I have to check it immediately.
9. It is difficult for me to stop activities that I really enjoy.
10. I have a hard time working on tasks that do not interest me.

Optimism

1. No matter what happens, everything works out in the end.
2. I believe that people have good intentions.
3. I consider struggles to be temporary.
4. I can confidently face my problems.
5. It is difficult for me to be positive about life. *
6. I believe that the world is generally an unsafe place.*
7. If things go wrong, I am to blame*
8. I expect things to go wrong*
9. I think positively about my future.*
10. I maintain positivity even when others around me are not.*

Grit

1. My daily life consists of things that keep me interested.
2. Difficulties do not discourage me
3. I fully dedicate myself to tasks I am assigned to.
4. I tend to give up even when presented with multiple challenges.
5. Even if something is hard, I will keep trying at it.
6. I stay committed to a task, even when it's overwhelming.
7. I will complete a task even if I do not understand its meaning or purpose.

Fear and Worry Response

1. For the majority of my day I am overly alert and on guard.
2. I avoid activities because I expect something bad will happen.
3. I am easily alarmed and jumpy.
4. It is tough for me to mentally disconnect from online media.
5. I worry a lot about situations, events, or people that do not matter to me.
6. I cannot stop thinking about criticism or setbacks I have encountered.
7. I remain highly frustrated when I think back on recent challenges.
8. My concerns overburden me.
9. I can maintain a calm demeanor when under pressure.
10. It is easy for me to let go of worrisome thoughts.
11. While finishing a task, I begin to worry in anticipation about all remaining tasks.
12. I do not worry about situations or events that are out of my control.
13. Worry prevents me from concentrating on crucial tasks throughout the day.
14. I lose sleep because of my worries.

*indicates items that are reverse scored
