# Supplementary material for: The cognitive adaptability and resiliency employment screener (CARES): tool development and testing
Source: Front Psychiatry. 2023 Sep 29;14:1254147. doi: 10.3389/fpsyt.2023.1254147 (PMC10570752; doi:10.3389/fpsyt.2023.1254147)
Supplement: Supplementary file 1 [file Data_Sheet_1.zip › Appendix 2_CARES Descriptives_Phase 1.docx]

| **Appendix 2.** Descriptives of Phase 1 Items | | | |  |  |  |
| --- | --- | --- | --- | --- | --- | --- |
|  | Mean | SD | Min | Max | Skew | Kurtosis |
| er_1 | 4.72 | 1.63 | 0 | 6 | -1.57 | 1.65 |
| er_2 | 3.01 | 1.59 | 0 | 6 | -0.13 | -0.68 |
| er_3 | 2.67 | 1.61 | 0 | 6 | 0.14 | -0.77 |
| er_4 | 2.06 | 1.68 | 0 | 6 | 0.57 | -0.65 |
| er_5 | 4.71 | 1.37 | 0 | 6 | -1.3 | 1.47 |
| er_6 | 1.83 | 1.49 | 0 | 6 | 0.75 | 0.06 |
| er_7 | 3.89 | 1.52 | 0 | 6 | -0.53 | -0.28 |
| er_8 | 2.06 | 1.63 | 0 | 6 | 0.58 | -0.44 |
| ei_1 | 0.93 | 1.21 | 0 | 6 | 2.02 | 4.82 |
| ei_2 | 2.49 | 1.74 | 0 | 6 | 0.31 | -0.89 |
| ei_3 | 4.75 | 1.26 | 0 | 6 | -1.49 | 2.61 |
| ei_4 | 3.42 | 1.53 | 0 | 6 | -0.24 | -0.50 |
| ei_5 | 2.27 | 1.68 | 0 | 6 | 0.38 | -0.80 |
| ei_6 | 3.00 | 1.69 | 0 | 6 | -0.06 | -0.85 |
| ei_7 | 1.06 | 1.26 | 0 | 6 | 1.67 | 3.16 |
| ei_8 | 3.09 | 1.58 | 0 | 6 | -0.12 | -0.60 |
| ea_1 | 1.54 | 1.51 | 0 | 6 | 0.98 | 0.25 |
| ea_2 | 1.52 | 1.39 | 0 | 6 | 0.95 | 0.33 |
| ea_3 | 3.66 | 1.51 | 0 | 6 | -0.34 | -0.49 |
| ea_4 | 2.32 | 1.47 | 0 | 6 | 0.22 | -0.54 |
| ea_5 | 3.45 | 1.80 | 0 | 6 | -0.35 | -0.87 |
| ea_6 | 2.36 | 1.67 | 0 | 6 | 0.25 | -0.84 |
| ea_7 | 1.49 | 1.53 | 0 | 6 | 1.06 | 0.55 |
| ea_8 | 1.15 | 1.34 | 0 | 6 | 1.29 | 1.25 |
| ccfq_1 | 5.47 | 1.02 | 0 | 6 | -3.33 | 13.50 |
| ccfq_2 | 5.36 | 1.07 | 0 | 6 | -2.87 | 10.10 |
| ccfq_3 | 4.66 | 1.38 | 0 | 6 | -1.33 | 1.65 |
| ccfq_4 | 5.20 | 1.10 | 0 | 6 | -2.23 | 6.58 |
| ccfq_5 | 5.23 | 1.12 | 0 | 6 | -2.42 | 7.30 |
| ccfq_6 | 5.23 | 1.08 | 0 | 6 | -2.33 | 7.13 |
| ccfq_7 | 3.93 | 1.67 | 0 | 6 | -0.56 | -0.6 |
| ccfq_8 | 4.90 | 1.24 | 0 | 6 | -1.46 | 2.46 |
| ccfq_9 | 5.40 | 1.01 | 0 | 6 | -2.94 | 11.13 |
| ccfq_10 | 2.40 | 1.74 | 0 | 6 | 0.31 | -0.92 |
| neuroticism_1 | 5.02 | 1.31 | 0 | 6 | -2.05 | 4.5 |
| neuroticism_2 | 4.24 | 1.46 | 0 | 6 | -0.67 | -0.31 |
| neuroticism_3 | 1.58 | 1.58 | 0 | 6 | 1.01 | 0.30 |
| neuroticism_4 | 3.16 | 1.90 | 0 | 6 | -0.13 | -1.14 |
| neuroticism_5 | 2.01 | 1.54 | 0 | 6 | 0.78 | 0.08 |
| neuroticism_6 | 1.47 | 1.53 | 0 | 6 | 1.16 | 0.71 |
| neuroticism_7 | 1.57 | 1.47 | 0 | 6 | 0.86 | 0.12 |
| neuroticism_8 | 3.14 | 1.99 | 0 | 6 | -0.01 | -1.24 |
| neuroticism_9 | 3.32 | 1.86 | 0 | 6 | -0.22 | -1.05 |
| neuroticism_10 | 1.84 | 1.59 | 0 | 6 | 0.68 | -0.36 |
| optimism_1 | 5.18 | 1.16 | 0 | 6 | -2.11 | 5.29 |
| optimism_2 | 4.82 | 1.31 | 0 | 6 | -1.27 | 1.47 |
| optimism_3 | 5.26 | 1.13 | 0 | 6 | -2.32 | 6.36 |
| optimism_4 | 5.32 | 1.03 | 0 | 6 | -2.49 | 8.24 |
| optimism_5 | 4.88 | 1.47 | 0 | 6 | -1.63 | 2.15 |
| optimism_6 | 3.82 | 1.76 | 0 | 6 | -0.42 | -0.84 |
| optimism_7 | 4.18 | 1.59 | 0 | 6 | -0.66 | -0.30 |
| optimism_8 | 4.70 | 1.56 | 0 | 6 | -1.25 | 0.76 |
| optimism_9 | 5.52 | 1.00 | 0 | 6 | -3.11 | 11.62 |
| optimism_10 | 5.38 | 1.07 | 0 | 6 | -2.65 | 8.66 |
| grit_1 | 4.76 | 1.39 | 0 | 6 | -1.37 | 1.66 |
| grit_2 | 4.92 | 1.46 | 0 | 6 | -1.76 | 2.66 |
| grit_3 | 5.46 | 0.97 | 0 | 6 | -3.16 | 12.94 |
| grit_4 | 1.21 | 1.51 | 0 | 6 | 1.64 | 2.24 |
| grit_5 | 5.44 | 0.98 | 0 | 6 | -3.04 | 12.02 |
| grit_6 | 5.26 | 1.06 | 0 | 6 | -2.38 | 7.52 |
| grit_7 | 3.74 | 1.77 | 0 | 6 | -0.51 | -0.73 |
| worry_1 | 4.61 | 1.39 | 0 | 6 | -1.26 | 1.39 |
| worry_2 | 1.19 | 1.32 | 0 | 6 | 1.48 | 2.18 |
| worry_3 | 2.16 | 1.73 | 0 | 6 | 0.48 | -0.77 |
| worry_4 | 1.60 | 1.55 | 0 | 6 | 0.92 | 0.04 |
| worry_5 | 1.87 | 1.65 | 0 | 6 | 0.69 | -0.44 |
| worry_6 | 1.73 | 1.60 | 0 | 6 | 0.81 | -0.20 |
| worry_7 | 1.26 | 1.35 | 0 | 6 | 1.29 | 1.38 |
| worry_8 | 1.35 | 1.37 | 0 | 6 | 1.13 | 0.81 |
| worry_9 | 1.12 | 1.33 | 0 | 6 | 1.66 | 2.82 |
| worry_10 | 1.58 | 1.48 | 0 | 6 | 1.05 | 0.67 |
| worry_11 | 2.18 | 1.68 | 0 | 6 | 0.52 | -0.68 |
| worry_12 | 2.34 | 1.78 | 0 | 6 | 0.40 | -0.83 |
| worry_13 | 2.02 | 1.79 | 0 | 6 | 0.65 | -0.69 |
| worry_14 | 1.51 | 1.59 | 0 | 6 | 1.03 | 0.17 |
