# Supplementary material for: The cognitive adaptability and resiliency employment screener (CARES): tool development and testing
Source: Front Psychiatry. 2023 Sep 29;14:1254147. doi: 10.3389/fpsyt.2023.1254147 (PMC10570752; doi:10.3389/fpsyt.2023.1254147)
Supplement: Supplementary file 1 [file Data_Sheet_1.zip › Appendix 3_CARES_Descriptives_Phase 2.docx]

| **Appendix 3.** Descriptives of Phase 2 Items | | | |  |  |  |
| --- | --- | --- | --- | --- | --- | --- |
|  | Mean | SD | Min | Max | Skew | Kurtosis |
| er_1 | 4.67 | 1.66 | 0 | 6 | -1.57 | 1.58 |
| er_2 | 2.93 | 1.61 | 0 | 6 | -0.04 | -0.70 |
| er_3 | 2.57 | 1.61 | 0 | 6 | 0.23 | -0.70 |
| er_4 | 1.93 | 1.64 | 0 | 6 | 0.69 | -0.45 |
| er_5 | 4.73 | 1.39 | 0 | 6 | -1.30 | 1.42 |
| er_6 | 1.84 | 1.5 | 0 | 6 | 0.73 | -0.01 |
| er_7 | 3.86 | 1.53 | 0 | 6 | -0.50 | -0.30 |
| er_8 | 1.88 | 1.54 | 0 | 6 | 0.71 | -0.18 |
| ei_1 | 0.91 | 1.17 | 0 | 6 | 2.10 | 5.45 |
| ei_2 | 2.45 | 1.73 | 0 | 6 | 0.38 | -0.81 |
| ei_3 | 4.77 | 1.23 | 0 | 6 | -1.62 | 3.27 |
| ei_4 | 3.41 | 1.53 | 0 | 6 | -0.23 | -0.53 |
| ei_5 | 2.24 | 1.63 | 0 | 6 | 0.34 | -0.82 |
| ei_7 | 1.02 | 1.28 | 0 | 6 | 1.83 | 3.79 |
| ei_8 | 3.11 | 1.53 | 0 | 6 | -0.13 | -0.55 |
| ea_1 | 1.56 | 1.54 | 0 | 6 | 1.06 | 0.39 |
| ea_2 | 1.52 | 1.29 | 0 | 6 | 0.97 | 0.70 |
| ea_3 | 3.64 | 1.49 | 0 | 6 | -0.37 | -0.37 |
| ea_4 | 2.37 | 1.47 | 0 | 6 | 0.31 | -0.42 |
| ea_6 | 2.36 | 1.64 | 0 | 6 | 0.31 | -0.71 |
| ea_7 | 1.48 | 1.50 | 0 | 6 | 1.05 | 0.59 |
| ea_8 | 1.07 | 1.25 | 0 | 6 | 1.40 | 1.86 |
| ccfq_1 | 5.49 | 0.96 | 0 | 6 | -3.33 | 14.09 |
| ccfq_2 | 5.35 | 1.03 | 0 | 6 | -2.92 | 10.88 |
| ccfq_3 | 4.62 | 1.38 | 0 | 6 | -1.28 | 1.49 |
| ccfq_4 | 5.16 | 1.14 | 0 | 6 | -2.03 | 5.17 |
| ccfq_5 | 5.28 | 1.04 | 0 | 6 | -2.40 | 7.70 |
| ccfq_6 | 5.23 | 1.08 | 0 | 6 | -2.19 | 6.16 |
| ccfq_7 | 3.97 | 1.62 | 0 | 6 | -0.56 | -0.63 |
| ccfq_8 | 4.92 | 1.21 | 0 | 6 | -1.45 | 2.40 |
| ccfq_9 | 5.42 | 0.99 | 0 | 6 | -2.91 | 11.14 |
| ccfq_10 | 2.41 | 1.72 | 0 | 6 | 0.35 | -0.82 |
| neuroticism_1 | 5.03 | 1.26 | 0 | 6 | -1.97 | 4.27 |
| neuroticism_2 | 4.32 | 1.41 | 0 | 6 | -0.70 | -0.17 |
| neuroticism_3 | 1.43 | 1.51 | 0 | 6 | 1.16 | 0.80 |
| neuroticism_6 | 1.54 | 1.57 | 0 | 6 | 1.06 | 0.40 |
| neuroticism_7 | 1.51 | 1.46 | 0 | 6 | 0.93 | 0.17 |
| neuroticism_9 | 3.35 | 1.83 | 0 | 6 | -0.22 | -0.99 |
| neuroticism_10 | 1.74 | 1.56 | 0 | 6 | 0.81 | -0.09 |
| optimism_1 | 5.16 | 1.19 | 0 | 6 | -2.01 | 4.71 |
| optimism_2 | 4.81 | 1.28 | 0 | 6 | -1.18 | 1.19 |
| optimism_3 | 5.28 | 1.10 | 0 | 6 | -2.33 | 6.48 |
| optimism_4 | 5.33 | 1.00 | 0 | 6 | -2.30 | 7.15 |
| optimism_5 | 4.82 | 1.57 | 0 | 6 | -1.58 | 1.79 |
| optimism_6 | 3.98 | 1.74 | 0 | 6 | -0.51 | -0.73 |
| optimism_8 | 4.76 | 1.56 | 0 | 6 | -1.29 | 0.75 |
| optimism_9 | 5.55 | 0.95 | 0 | 6 | -3.07 | 11.38 |
| optimism_10 | 5.41 | 1.04 | 0 | 6 | -2.6 | 8.20 |
| grit_1 | 4.73 | 1.39 | 0 | 6 | -1.4 | 1.84 |
| grit_2 | 4.94 | 1.43 | 0 | 6 | -1.85 | 3.14 |
| grit_3 | 5.48 | 0.92 | 0 | 6 | -3.14 | 13.29 |
| grit_4 | 1.15 | 1.48 | 0 | 6 | 1.79 | 2.87 |
| grit_5 | 5.46 | 0.93 | 0 | 6 | -3.02 | 12.23 |
| grit_6 | 5.28 | 1.03 | 0 | 6 | -2.34 | 7.29 |
| worry_1 | 4.61 | 1.36 | 0 | 6 | -1.18 | 1.12 |
| worry_2 | 1.10 | 1.23 | 0 | 6 | 1.58 | 2.93 |
| worry_3 | 2.15 | 1.71 | 0 | 6 | 0.56 | -0.62 |
| worry_4 | 1.51 | 1.48 | 0 | 6 | 1.03 | 0.42 |
| worry_5 | 1.87 | 1.64 | 0 | 6 | 0.65 | -0.50 |
| worry_6 | 1.74 | 1.57 | 0 | 6 | 0.82 | -0.11 |
| worry_7 | 1.26 | 1.35 | 0 | 6 | 1.29 | 1.37 |
| worry_8 | 1.35 | 1.33 | 0 | 6 | 1.13 | 1.14 |
| worry_9 | 1.14 | 1.34 | 0 | 6 | 1.73 | 3.16 |
| worry_10 | 1.65 | 1.50 | 0 | 6 | 0.96 | 0.38 |
| worry_11 | 2.17 | 1.68 | 0 | 6 | 0.53 | -0.69 |
| worry_13 | 1.87 | 1.68 | 0 | 6 | 0.83 | -0.24 |
| worry_14 | 1.49 | 1.55 | 0 | 6 | 1.04 | 0.22 |
