# Supplementary material for: The cognitive adaptability and resiliency employment screener (CARES): tool development and testing
Source: Front Psychiatry. 2023 Sep 29;14:1254147. doi: 10.3389/fpsyt.2023.1254147 (PMC10570752; doi:10.3389/fpsyt.2023.1254147)
Supplement: Supplementary file 1 [file Data_Sheet_1.zip › Appendix 4_CARES_Retained Items.docx]

**CARES (67 items retained)**

Factor 1: Psychological Perseverance and Agility (PPA)

1. It only takes a few moments to calm myself following an argument.
2. I tend to think more logically instead of emotionally.
3. I am able to remain calm following any stressful situation.
4. I quickly recover from being startled/ alarmed.
5. I care about how my decisions will impact others.
6. I rely on facts over intuition.
7. I am able to prioritize and focus on important tasks.
8. When I feel sad, I can still do the tasks that need to be done
9. After I resolve a problem, I do not obsess about it afterwards.
10. I am comfortable multitasking.
11. I am able to set aside unrelated thoughts while focusing on a current task/conversation.
12. I am able to apply past successful strategies to current difficulties.
13. I am flexible and comfortable with rapid changes.
14. If I do not succeed on my first attempt, I find other solutions.
15. I do not let myself become “stuck” on past events.
16. I can focus on my work even when I am in a noisy environment.
17. No matter what happens, everything works out in the end.
18. I believe that people have good intentions.
19. I consider struggles to be temporary.
20. I can confidently face my problems.
21. I think positively about my future.
22. I maintain positivity even when others around me are not.
23. My daily life consists of things that keep me interested.
24. Difficulties do not discourage me
25. I fully dedicate myself to tasks I am assigned to.
26. Even if something is hard, I will keep trying at it.
27. I stay committed to a task, even when it's overwhelming.
28. For the majority of my day I am overly alert and on guard.
29. I can maintain a calm demeanor when under pressure.
30. It is easy for me to let go of worrisome thoughts.

Factor 2: Rumination and Emotion Lingering (REL)

1. I have a tough time speaking up for myself.
2. When an opportunity is given to me to share how I feel, I prefer not to.
3. I become easily overwhelmed when provided too many tasks.
4. Work interruptions tend to upset me.
5. I find it difficult to relax after viewing disturbing content on my television or computer.
6. Whenever I feel excited, it is difficult for me to focus.
7. It is difficult for me to not get overwhelmed.
8. I consider myself as an emotional person.
9. I tend to remain upset for long periods of time following an argument.
10. I have a difficult time adjusting to last minute changes.
11. I cannot switch tasks when I am in deep concentration.
12. I get distracted by my thoughts.
13. I only work on tasks that I find rewarding.
14. I get bored doing repetitive tasks.
15. It is difficult for me to stop activities that I really enjoy.
16. I have a hard time working on tasks that do not interest me.
17. It is difficult for me to be positive about life.
18. I believe that the world is generally an unsafe place.
19. I expect things to go wrong.
20. I tend to give up even when presented with multiple challenges.
21. I avoid activities because I expect something bad will happen.
22. I am easily alarmed and jumpy.
23. It is tough for me to mentally disconnect from online media.
24. I worry a lot about situations, events, or people that do not matter to me.
25. I cannot stop thinking about criticism or setbacks I have encountered.
26. I remain highly frustrated when I think back on recent challenges.
27. My concerns overburden me.
28. While finishing a task, I begin to worry in anticipation about all remaining tasks.
29. Worry prevents me from concentrating on crucial tasks throughout the day.
30. I lose sleep because of my worries.

Factor 3: Expressiveness and Sociability (ESc)

1. I prefer not to tell others what I am feeling.*
2. I prefer not to tell others what I am thinking.*
3. I would consider myself as someone who shares feelings openly.
4. I am very open and inform others of my current feelings.
5. I am a person who is highly expressive about my emotions.
6. I have a tendency to express my emotions immediately.
7. It is easy for me to express my emotions.

*indicates items that are reverse scored
