# Supplementary material for: The cognitive adaptability and resiliency employment screener (CARES): tool development and testing
Source: Front Psychiatry. 2023 Sep 29;14:1254147. doi: 10.3389/fpsyt.2023.1254147 (PMC10570752; doi:10.3389/fpsyt.2023.1254147)
Supplement: Supplementary file 1 [file Data_Sheet_1.zip › Appendix 5. HTMT Table.docx]

Appendix 5. HTMT Analyses

|  | Factor 1 | Factor 2 | Factor 3 |
| --- | --- | --- | --- |
| Factor 1 | 1 |  |  |
| Factor 2 | 0.289 | 1 |  |
| Factor 3 | 0.318 | 0.254 | 1 |
